# Association Between Arterial Oxygen Saturation and Lung Ultrasound B-Lines After Competitive Deep Breath-Hold Diving

**DOI:** 10.3389/fphys.2021.711798

**Published:** 2021-08-04

**Authors:** Alexander Patrician, Frank Pernett, Angelica Lodin-Sundström, Erika Schagatay

**Affiliations:** ^1^Centre for Heart, Lung & Vascular Health, University of British Columbia, Okanagan, BC, Canada; ^2^Environmental Physiology Group, Department of Health Sciences, Mid Sweden University, Östersund, Sweden; ^3^Department of Nursing Science, Mid Sweden University, Sundsvall, Sweden; ^4^Swedish Winter Sports Research Centre, Department of Health Sciences, Mid Sweden University, Östersund, Sweden

**Keywords:** hypoxia, apnea, hypoxic syncope, blackout, pulmonary edema, barotrauma, injury, extreme environment

## Abstract

Breath-hold diving (freediving) is an underwater sport that is associated with elevated hydrostatic pressure, which has a compressive effect on the lungs that can lead to the development of pulmonary edema. Pulmonary edema reduces oxygen uptake and thereby the recovery from the hypoxia developed during freediving, and increases the risk of hypoxic syncope. We aimed to examine the efficacy of SpO_2_, via pulse-oximetry, as a tool to detect pulmonary edema by comparing it to lung ultrasound B-line measurements after deep diving. SpO_2_ and B-lines were collected in 40 freedivers participating in an international deep freediving competition. SpO_2_ was measured within 17 ± 6 min and lung B-lines using ultrasound within 44 ± 15 min after surfacing. A specific symptoms questionnaire was used during SpO_2_ measurements. We found a negative correlation between B-line score and minimum SpO_2_ (*r*_*s*_ = −0.491; *p* = 0.002) and mean SpO_2_ (*r*_*s*_ = −0.335; *p* = 0.046). B-line scores were positively correlated with depth (*r*_*s*_ = 0.408; *p* = 0.013), confirming that extra-vascular lung water is increased with deeper dives. Compared to dives that were asymptomatic, symptomatic dives had a 27% greater B-line score, and both a lower mean and minimum SpO_2_ (all *p* < 0.05). Indeed, a minimum SpO_2_ ≤ 95% after a deep dive has a positive predictive value of 29% and a negative predictive value of 100% regarding symptoms. We concluded that elevated B-line scores are associated with reduced SpO_2_ after dives, suggesting that SpO_2_ via pulse oximetry could be a useful screening tool to detect increased extra-vascular lung water. The practical application is not to diagnose pulmonary edema based on SpO_2_ – as pulse oximetry is inexact – rather, to utilize it as a tool to determine which divers require further evaluation before returning to deep freediving.

## Introduction

Breath-hold diving, also known as freediving, ranges from recreational and professional activities to a competitive sport with several disciplines for the maximal duration, distance, or depth performed on a single breath (Fitz-Clarke, 2018). Within each discipline, a specific combination of physiological factors determine performance (Schagatay, 2009, 2010, 2011; Bain et al., 2018).

Breath-hold diving is associated with an increased hydrostatic pressure that has a compressive effect on the body’s air-filled cavities. As such, the rise in ambient pressure compresses the lungs in accordance with Boyle’s law, thus reducing lung volume as the diver descends. The lungs are indeed compliant, but being delicate, exceeding the compliant threshold of the lung may lead to pulmonary edema (Lindholm et al., 2008), commonly referred to by divers as “lung squeeze.” Pulmonary edema is associated with the accumulation of fluid in the alveoli, thus preventing oxygen uptake and causing hypoxemia (Lindholm and Lundgren, 2009). When affected, divers present with symptoms of productive cough, dyspnea, chest tightness (Cialoni et al., 2012), hemoptysis, and exhibit desaturation with a reduction in pulmonary function tests (Linér and Andersson, 2008). However, it would be just before, or upon surfacing where divers are most vulnerable because of the risk of hypoxic syncope, or “blackout” (Lindholm, 2007). The presence of fluid in the lungs would reduce the diffusion of oxygen from the alveolar space into the pulmonary circulation, thereby restraining the recovery from a low arterial oxygen saturation upon surfacing. Deep freediving additionally requires strenuous effort, and exercise stresses the pulmonary capillaries (West, 2004) increasing the risk of fluid leakage and increasing the diffusion barrier. One lethal case is known from a breath-hold diving competition in 2013, where a diver surfaced after a deep dive but experienced a “blackout” and was unable to recover due to severe lung squeeze (Vestin, 2015).

Ultrasound B-lines, also referred to as ultrasound lung comets, has been found to provide an accurate estimation of extravascular lung water (Jambrik et al., 2004; Agricola et al., 2005) and are considered to be a reliable tool to evaluate pulmonary edema (Lichtenstein, 2014; Chiumello et al., 2016; Wang et al., 2018; Soldati et al., 2019). B-lines have previously been measured in breath-hold divers, and depth does indeed appear to be a potent contributor to increase the B-line score (Frassi et al., 2008; Boussuges et al., 2011; Lambrechts et al., 2011) and pulmonary gas exchange impairment (Patrician et al., 2021). However, ultrasound imaging is not readily available outside of the research and clinical community. Therefore, it would be ideal to determine the efficacy of an easier and simpler tool for post-dive evaluation in freedivers, which provides insight into the potential of extravascular lung water. Preliminary data collected from 100 competitive dives have suggested that monitoring peripheral oxygen saturation (SpO_2_) in the 20 min following the dive could be a tool to detect pulmonary edema/lung squeeze, as it was associated with reported and observed symptoms (Schagatay et al., 2015).

Our study sought to test the primary hypothesis that the impairment of SpO_2_ recovery following deep breath-hold diving would be associated with elevated B-line score, an accepted and informative index of extravascular lung water. To examine this hypothesis, we studied breath-hold divers during a depth world championship.

## Materials and Methods

### Participants

A total of 40 freedivers, 28 males and 12 females, with a mean ± SD age of 35 ± 7 years; 177 ± 7 cm height and 70 ± 8 kg weight and body mass index 22 ± 2 kg/m^2^ volunteered to be in the study. All participants provided verbal informed consent, following written and oral information on the study and protocol. The study was reviewed by the local research ethics board and conformed to the Declaration of Helsinki. The study was also approved by and conducted in conjunction with the competition organizing committee.

While the measurements during this study likely capture maximal levels of physiological strain and provide valuable insight into the limits of the human body during freediving, the nature of a professional competition unavoidably led to variability in the timing of assessments. The competition dives were performed in three different dive disciplines: constant weight with fins (CWT), constant weight without fins (CNF), and free immersion (FIM) over 6 days of competition. A review of these disciplines has been covered elsewhere (Schagatay, 2011).

### Procedures

Following each dive, SpO_2_ and heart rate were recorded in 15-s intervals for 2 min via pulse oximetry (Nonin Onyx Vantage 9590) within 20 ± 11 min (range:10–45 min) of surfacing. Pulse oximetry data was collected in a finger, in the upright position, and only recorded when the oximetry signal was strong, as indicated by a bright green light signal. If the signal strength was sub-optimal (e.g., due to cold fingers), divers were given time to warm up before re-assessing. Ultrasound B-lines were collected in the supine position [within 45 ± 18 min (range: 18–85 min) of surfacing] and performed using 2-dimensional ultrasonic imaging, with a convex transducer 2–5 MHz (M-Turbo ultrasound system, FUJIFILM SonoSite Inc., Bothell, WA, United States). Bilateral imaging of the hemithorax (parasternal, mid-clavicular, anterior axillary line, and mid-axillary) from the second to fourth (fifth on the right side) intercostal spaces were performed, culminating in 28 zones. A B-line was defined as an echogenic, coherent, and wedge-shaped signal with a narrow origin in the near field of the image, spreading from the pleural line to the further border of the screen, which moves in concert with lung sliding (Lichtenstein, 2014). The same investigator (AL-S) performed all B-line measurements. The total number of B-lines in each zone was counted and summed to provide a total B-line score. For situations when the entire zone was full of B-lines (i.e., white-out) a maximum of 20 was assigned. B-lines are an index of extravascular lung fluid, which has been performed previously with high sensitivity and intra-patient reliability to fluid visible using radiographic imaging (Jambrik et al., 2004; Picano et al., 2006; Boussuges et al., 2011; Lambrechts et al., 2011). As part of the evaluation, the participant was also asked for specific symptoms. Symptoms considered relevant were cough, mild-severe chest discomfort, tightness and/or irritation along the respiratory tract (i.e., throat to lungs) and hemoptysis. The participant was considered symptomatic if one or more of these symptoms was present. Post-dive evaluation and measurements were performed on the support boat for the competition.

In a subgroup of divers (*n* = 6), baseline B-line measurements were conducted before the competition, and with at least 12 h without diving activity.

Given the unavoidable range in measurement times (i.e., time from surfacing to measurement); a time-specific analysis was conducted – using pulse oximetry within 30 min as a cut-off. A total of 50 dives: 10 CNF, 21 CWT, and 19 FIM at a mean depth of 76 ± 21 m (range: 40–122 m) with pulse oximetry within 18 ± 6 min (range: 6–30 min) and B-lines within 41 ± 15 min (range: 17–82 min) were included. For correlational analysis (see section “Statistical analysis”), only the first dive for each diver were included (i.e., 36 dives), to ensure the independence of cases since each diver competed over several days.

As part of the competition, supplemental O_2_ was available for divers upon surfacing, it was used in 36 dives (72%) and SpO_2_ was measured 26 ± 11 min (range: 10–50 min) after exposure.

### Statistical Analysis

Data was assessed for normality using Kolmogorov-Smirnov tests (IBM SPSS, Statistics, United States). Differences from baseline B-line score, the right vs. left lung and symptomatic vs. asymptomatic dives were evaluated using the Mann-Whitney test. Effect size (*R*) was calculated using the Mann-Whitney *Z* score, divided by the square root of the sample size (*n*). Correlations were evaluated using Spearman’s (*r*_*s*_) correlational analysis. All data is presented as mean ± SD and significance was assumed at *p* < 0.05.

## Results

### B-Line Score and SpO_2_

**FIGURE 1:**
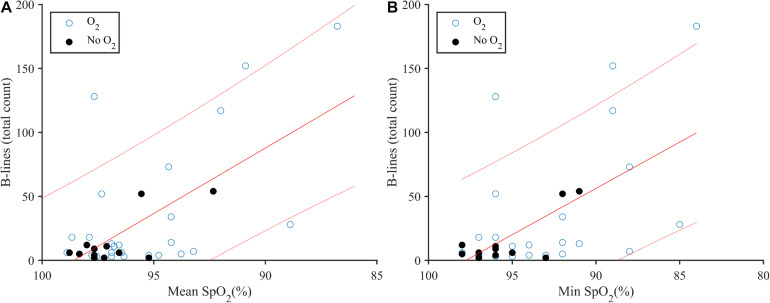
**(A)** Correlation plot of B-lines and mean SpO_2_ following deep breath-hold diving (*r*_*s*_ = −0.335, *p* = 0.046) for *n* = 36. Use of supplemental oxygen at the end of the dive is indicated by used (O_2_; open circles) and not used (No O_2_, closed circles). **(B)** B-lines and minimum (Min) SpO_2_ following deep breath-hold diving (*r*_*s*_ = −0.491, *p* = 0.002) for *n* = 36. Linear regression (solid line) with 90% confidence intervals (dotted line).

B-line score was negatively correlated with mean SpO_2_ (*r*_*s*_ = −0.335, *p* = 0.046; Figure 1A) and minimum SpO_2_ (*r*_*s*_ = −0.491, *p* = 0.002; Figure 1B). Across all zones, there were on average 35 ± 49 B-lines, with a mean and minimum SpO_2_ of 96.4 ± 2.8 and 93.7 ± 4.3%, respectively.

**FIGURE 2:**
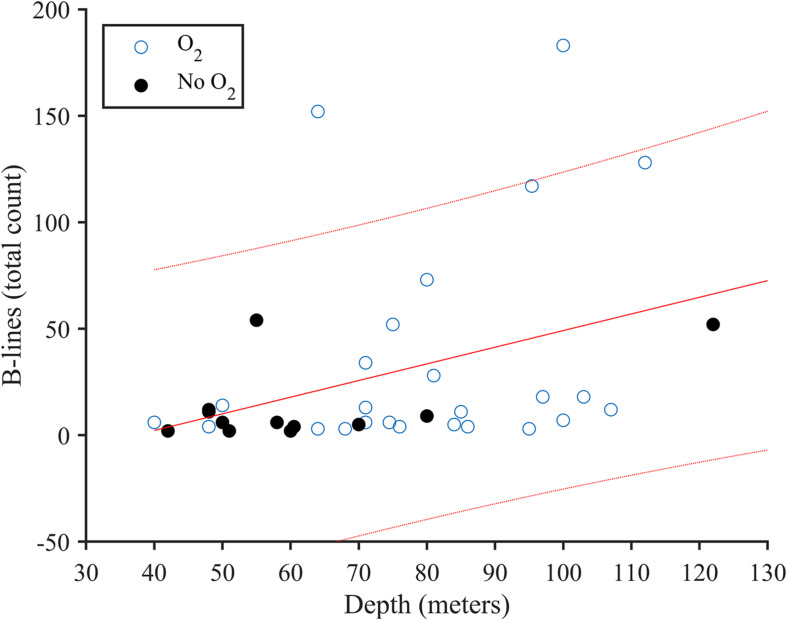
Correlation plot of B-lines and diving depth (*r*_*s*_ = 0.408, *p* = 0.013) for *n* = 36. Use of supplemental oxygen at the end of the dive is indicated by used (O_2_; open circles) and not used (No O_2_, closed circles). Linear regression (solid line) with 90% confidence intervals (dotted line).

Post-dive B-line score was positively correlated with depth (*r*_*s*_ = 0.408, *p* = 0.013; Figure 2). When intercostal regions II-IV were evaluated between sides (i.e., given intercostal region V is confounded by the heart on the left side), the right lung appeared to have a higher B-line score (19 ± 26 B-lines) following the dive compared to the left lung (9 ± 18 B-lines; *U* = 784.5, *p* = 0.001, *R* = 0.322).

### Symptoms

**FIGURE 3:**
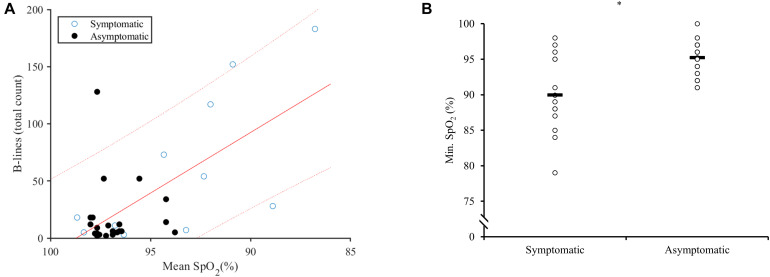
**(A)** Correlation plot of respiratory symptoms and the relationship between B-lines and mean SpO_2_. Symptomatic (open circles) and asymptomatic (closed circles). Linear regression (solid line) with 90% confidence intervals (dotted line). **(B)** Dot plot of minimum SpO_2_ in symptomatic divers, versus asymptomatic divers. Horizontal line represents the means at 89.9 ± 5.4% for symptomatic and 95.2 ± 2.3% for asymptomatic divers. * Indicates *p* < 0.05 between groups.

When B-line and SpO_2_ were grouped based on symptoms, symptomatic dives showed significantly lower SpO_2_, higher B-line score and greater depth (Figure 3 and Table 1). Mean SpO_2_ also correlated positively with symptoms (*r*_*s*_ = −0.421, *p* = 0.010), and with depth (*r*_*s*_ = −0.381, *p* = 0.022). Minimum SpO_2_ was negatively correlated with both symptoms (*r*_*s*_ = −0.468, *p* = 0.004) and depth (*r*_*s*_ = −0.347, *p* = 0.038).

**TABLE 1:** Symptomatology on post-dive measurements when pulse oximetry was collected within 30 min.

	**Asymptomatic**	**Symptomatic**	**U**	***p*-value**	**Effect size**
Dives (count)	35	15			
B-lines (total count)	18 ± 27	67 ± 63*	118	0.002	0.433
TAS-B-lines (min)	43.2 ± 15.6	45.4 ± 14.7	239.5	0.624	0.069
Mean SpO_2_	96.7 ± 1.7	93.8 ± 3.5*	133	0.006	0.388
Min. SpO_2_	95.2 ± 2.3	89.9 ± 5.4*	108	0.001	0.467
HR (bpm)	92 ± 12	98 ± 12	193	0.141	0.208
TAS-P.Ox (min)	16.1 ± 6.1	20.0 ± 5.4*	169	0.047	0.281
Depth (m)	71 ± 20	88 ± 18*	135	0.007	0.382

*Mean ± SD.*

TAS, time after surfacing (i.e., the time between surfacing and measurement of B-lines and pulse oximetry [P.Ox], respectively).

*p < 0.05.

### Baseline and Post-Dive B-Lines

**FIGURE 4:**
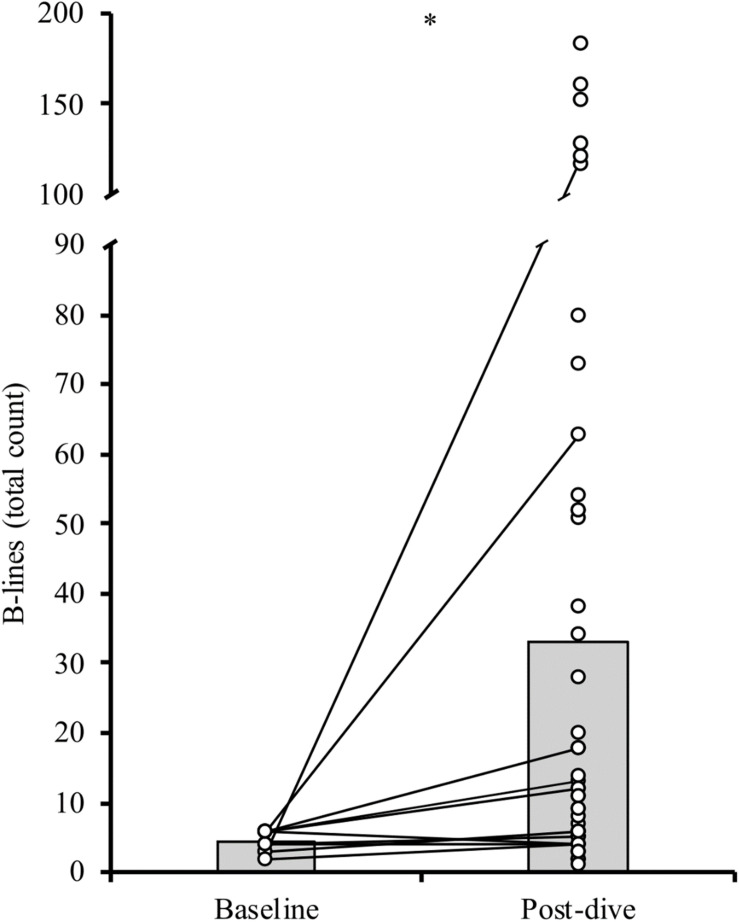
Individual (circles) and mean (bar) B-line scores at baseline and following the competitive dives. Solid lines connect baseline with post-dive values in participants with a baseline score (*n* = 6). ^∗^*p* < 0.05.

There was an increase in mean B-line scores from baseline at 5 ± 2 (range: 2–6; *n* = 6) to post-dive at 33 ± 46 (range: 1–183; *n* = 50; *U* = 65.6, *p* = 0.012, *R* = 0.308; Figure 4). Males (35 dives) demonstrated higher B-line scores (41 ± 50 B-lines) compared to females (15 dives; 9 ± 9 B-lines; *U* = 340.5, *p* = 0.01, *R* = 0.362). However, males had deeper dives (81 ± 20 m) than females (60 ± 13 m; *U* = 131.5, *p* = 0.001, *R* = 0.460).

## Discussion

Our main finding is that SpO_2_ measured within 30 min of surfacing correlates inversely to the prevalence of B-lines. This shows that impairments in the recovery of SpO_2_ during this period could reflect an excess of extra-pulmonary lung water. This relationship becomes further apparent as lower SpO_2_ was associated with respiratory symptoms. Aligning with our hypothesis, the measurement of pulse oximetry shortly after a dive is a simple and valuable tool for breath-hold divers for identifying possible lung harm. Indeed, many athletes, coaches, and organizers have begun incorporating pulse oximetry monitoring; however, this is the first study to establish its correlation with extravascular lung water during an actual competition, along with a specific time for measurement.

The positive correlation between depth and B-lines score aligns with earlier studies (Frassi et al., 2008; Lambrechts et al., 2011), and those demonstrating an increase in the incidence of pulmonary edema, via reduced spirometric performance (Linér and Andersson, 2008). Depth is also associated with an increase in pulmonary edema-related symptoms (Lindholm et al., 2008; Cialoni et al., 2012). However, as also shown in our data, some susceptible individuals may present symptoms after relatively shallow dives (Cialoni et al., 2012).

Aligning with Linér and colleagues, our data shows a negative correlation between depth and SpO_2_ (Linér and Andersson, 2008). While persistent post-dive hypoxemia may have multiple causes: (1) ventilation/perfusion mismatch due to atelectasis (Fahlman et al., 2009; Schipke et al., 2019). (2) increased right-to-left shunt; via increased strain on the right ventricle (Scherhag et al., 2005) and/or chronic pulmonary hypertension (Vestin, 2015), so if right atrial pressure becomes higher than left atrial pressure right-to-left shunting can occur (Layoun et al., 2017). A third cause could be diffusion limitation due to increased extravascular lung water (Picano et al., 2006; Boussuges et al., 2011; Picano and Pellikka, 2016). Finally, additional causes could be hypoventilation due to reduction in vital capacity, related to alveolar hemorrhage (Kiyan et al., 2001; Gouzi et al., 2007) or pulmonary barotrauma (Kiyan et al., 2001; Chung et al., 2010; Banham and Lippmann, 2019). Accordingly, persistent hypoxemia after diving, in a freediver, warrants a more in-depth analysis.

In addition, we found a clear difference in minimum SpO_2_ between divers with or without pulmonary symptoms. Indeed, all symptomatic dives had SpO_2_ below 95%, which further supports our main hypothesis that SpO_2_ could be useful to detect cases. B-line prevalence has a high range of reporting, varying from 14 to 31% (Frassi et al., 2008; Linér and Andersson, 2008; Cialoni et al., 2012) that can be explained by different approach (prospective or retrospective) and in our group, it was 15%. If we use the value of minimum SpO_2_ ≤ 95% after a deep dive, the positive predictive value is 29% and the negative predictive value is 100%. In terms of safety, it is better to have false positives that can be discarded with further investigation, than to let divers with pulmonary edema resume diving too early.

The unique finding that the right lung demonstrated an increased B-line score compared to the left lung (when matched for zones), is difficult to discern. However, there are some reports of unilateral swimming-induced pulmonary edema (Mahon et al., 2002; Lund et al., 2003), which could be related to increased perfusion on dependent zones of the lung. In terms of the biomechanics of propulsion, important differences exist between each discipline (Schagatay, 2011), but we did not find differences among disciplines. When freedivers reach the bottom plate, they have to make a change of direction to start swimming upward, this turn requires some thoracic torsion. This movement produces a change in thoracic compliance which can lead to a heterogeneous distribution of transpulmonary pressure (Cortes-Puentes et al., 2015). While we did not record which arm the divers turned with, if our cohort was primarily right-handed, it is intriguing to postulate that this might account for the uneven distribution of B-lines between right and left lung.

Our study thus confirms previous studies documenting an increase in B-lines after breath-hold diving (Frassi et al., 2008; Lambrechts et al., 2011). A relevant discussion revolves around how many B-lines are meaningful in diagnosing pulmonary edema in freedivers. Usually, they are not pronounced in the normal lung (Dietrich et al., 2016) and can be seen in other conditions, such as atelectasis (Dietrich et al., 2016), pneumonitis, and fibrosis that are unrelated to pulmonary edema (Volpicelli et al., 2012; Soldati et al., 2016). However, an increase in B-lines does not always coincide with pulmonary edema-related symptoms (Linér and Andersson, 2008). But, the notion that B-lines increase after diving and usually disappear in 24 h (Frassi et al., 2008) reinforces that it is an indirect measure of extra-vascular lung water which has been confirmed on other causes of pulmonary edema (Dietrich et al., 2016; Picano and Pellikka, 2016; Assaad et al., 2018).

With males diving deeper than females in the current study, it was not possible to evaluate whether there is a difference between sexes in B-line prevalence. However, with sex-related structural and functional differences of the lungs (Dominelli et al., 2019), different stroke volume during apnea (Magnani et al., 2018), and the unique hormonal role of estrogen on the pulmonary vasculature (Lahm et al., 2008), this is a valuable question and warrants further investigation.

Interestingly, not all deep dives lead to a substantial increase in B-lines and there is considerable variation in B-line score between divers. For example, some dives over 100 m had B-line scores ≤20, which is comparable to most of the 60-m dives. Many inter-individual differences could influence the incidence of B-lines. On one hand, there could be possible modifiable factors as a pre-dive warm-up, discipline, ascent/descent speed, use of glossopharyngeal insufflation (Chung et al., 2010), depth training (Cialoni et al., 2012), involuntary breathing movements at depth (Kiyan et al., 2001; Koehle et al., 2005; Lambrechts et al., 2011) and rib cage compliance. On the other hand, unmodifiable factors could be psychological stress, cold water (Koehle et al., 2005), lung volumes (Lindholm et al., 2008), residual volume to total lung capacity ratio (Ferretti et al., 2012), previous acute lung injuries (Carter et al., 2014), comorbidities and medications (Koehle et al., 2005), and genetic predisposition (Cialoni et al., 2015).

Safety is crucial in freediving, and there are no guidelines for safe return-to-dive practices regarding pulmonary edema. The overarching goal of the current study was to evaluate if pulse oximetry can act as a reliable marker of potential damage, to provide divers with information to enable them to evaluate their symptoms, and competition safety team to detect possibly affected divers. A pulse oximeter is an easy to use low-cost option for divers to be able to self-evaluate their condition post-dive. Efficient recovery of SpO_2_ is reliant upon efficient pulmonary gas exchange, which when impaired by fluid, prevents normal oxygenation, which is particularly dangerous for divers as they are severely hypoxic upon surfacing. Blood gas measurements of S_*a*_O_2_ and P_*a*_O_2_ in elite trained divers have been reported to drop well below 60% and 30 mmHg, respectively, during static apnea (Willie et al., 2015; Bain et al., 2017). Recently, the use of underwater near-infrared spectroscopy (NIRS) and pulse-oximetry reveals that even lower values develop after deep freediving (McKnight et al., 2021; Mulder and Schagatay, 2021). At such low levels of circulation oxygen, syncope can occur and the hypoxic blackout is not an uncommon occurrence (Pearn et al., 2015). Given the competitive progression of the sport of breath-hold diving, tools and guidelines aiming to improve diver safety are essential and important in keeping pace with the sport.

It is important to remark that there lacks a standardized definition of this type of pulmonary edema, induced by deep breath-hold diving. In the literature, there are a variety of terms in use, including squeeze (Lindholm and Lundgren, 2009; Schipke et al., 2019), pulmonary barotrauma, and immersion pulmonary edema (Moon et al., 2016; Kumar and Thompson, 2019). It has been suggested that a pathophysiological link exists between the pulmonary edema occurring during immersion and at high altitude. For example, high-resolution computed tomography imaging, albeit in a small cohort of patients (*n* = 4), suggests the heterogenetic blood flow response to hypoxia that occurs in high altitude pulmonary edema, may also be shared by those susceptible to immersion pulmonary edema (Lindholm et al., 2018). Furthermore, differences in lung structure and pulmonary lymphatics may also support a shared mechanism, especially in those susceptible to both high altitude pulmonary edema and immersion pulmonary edema (Carter et al., 2014). However, deep breath-hold diving imparts unique stress upon the pulmonary vasculature, since it is characterized by an increasing hydrostatic pressure that compresses both lung and thoracic cage (Fitz-Clarke, 2007). Due to differences in geometry and strain response (Plataki and Hubmayr, 2010; Andrikakou et al., 2016), the reduction in lung volume occurs at a quicker rate than the thoracic cage, and this interaction reduces intra-pleural pressure (Lai-Fook and Rodarte, 1991); thereby exaggerating the negative pleural pressure and increasing the hydrostatic pressure gradient. As the hydrostatic pressure gradient increases, any increase in the pulmonary artery pressure, due to the blood shift and hypoxic pulmonary vasoconstriction (Sylvester et al., 2012), could elevate pulmonary capillary pressure to the point of capillary stress failure (West et al., 1991). Together, this could lend support to distinct terminology of pulmonary edema because of breath-hold diving as a “depth-induced pulmonary edema.”

### Limitations

While our study is the first to correlate SpO_2_ with the number of lung ultrasound B-lines, it is important to note some limitations.

The small cohort of baseline lung b-line scores in the majority of participants does not allow us to conclude with certainty that the elevation in the B-line score correlates directly with the dive. However, our data aligns with baseline B-line scores in other breath-hold diving cohorts (Frassi et al., 2008; Boussuges et al., 2011; Lambrechts et al., 2011; Patrician et al., 2021). Additionally, given the study was non-interventional and all measures were collected alongside the competition, variability in timing was unavoidable. However, competition diving provides valuable insight into the current limits of physiology, since divers are highly motivated and performing single dives of maximal (or even supra-maximal) effort. By excluding values too far from the intended timing, we believe the measurements were within acceptable time limits for these circumstances. Furthermore, the nature of lung B-lines (extravascular lung water), and how they affect SpO_2_, would not disappear in one hour. Previous studies had shown that lung B-lines take around 24 h to disappear (Frassi et al., 2008; Cortellaro et al., 2017) and its reduction takes a few hours to occur (Picano et al., 2006). In that regard we will not expect substantial changes after 41 ± 15 min of surfacing, especially without any specific treatment (Dietrich et al., 2016), even oxygen inhalation does not seem to affect extra-vascular lung water (Pratali et al., 2012). Due to the lack of standardization of the dives (regarding warm-up, depth, and discipline), all correlations have to be seen with caution and our findings require confirmation in a more controlled setting.

Regarding SpO_2_, it is known that readings can be inaccurate when compared with SaO_2_ from blood gases (Wilson et al., 2010; Nitzan et al., 2014). For example, the used device has reported inaccuracy of 3.29% (95% CI 2.39–4.20%) during moderate hypoxic conditions (Ross et al., 2013). Additionally, vasoconstriction can also affect the readings (Wilson et al., 2010), but its effects last only 60 s on simulated dives (Heistad et al., 1968); on the other hand, sympathetic nerve activity returns to normal values within seconds after resuming breathing and arterial blood pressure returns rapidly to normal values once respiration is resumed (Ferrigno et al., 1997). As we measured SpO_2_ within 18 ± 6 min after surfacing and avoided recording from cold fingers/hands, we are confident in mediating the confounding influence of peripheral vasoconstriction. Therefore, we believe that persistent desaturation within 30 min will show real cases instead of false positives. However, as SpO_2_ is a surrogate for SaO_2_ and can be limited immediately post-dive, confirmation of these findings with arterial blood gas sampling is an important next step.

## Conclusion

SpO_2_ after dives was associated with ultrasound lung B-lines score, suggesting that SpO_2_ via pulse oximetry could be a useful tool, when measured within 30 min after surfacing, taking into account the intrinsic inaccuracy of the device. Ultrasound lung B-lines correlates with diving depth, confirming that extra-vascular lung water increases with deeper dives. Our findings can be important to increase freediving safety, by identifying injured divers to prevent them from continuing without proper recovery. Our data support the use of a SpO_2_ < 95% to identify possibly injured divers for further medical evaluation. Failure to identify these divers increases the risk of hypoxic syncope. This proof of concept study requires further research to evaluate its practical application.

## Data Availability Statement

The raw data supporting the conclusions of this article will be made available by the authors upon reasonable request.

## Ethics Statement

The study was reviewed by the local research ethics board and conformed to the Declaration of Helsinki. The study was also approved by and conducted in conjunction with the competition organizing committee.

## Author Contributions

AL-S and ES contributed to the conception of the study. AP, AL-S and ES contributed to the data acquisition. AP, FP and ES contributed to data analysis and manuscript writing and review. All authors approved the submitted version.

## Conflict of Interest

The authors declare that the research was conducted in the absence of any commercial or financial relationships that could be construed as a potential conflict of interest.

## Publisher’s Note

All claims expressed in this article are solely those of the authors and do not necessarily represent those of their affiliated organizations, or those of the publisher, the editors and the reviewers. Any product that may be evaluated in this article, or claim that may be made by its manufacturer, is not guaranteed or endorsed by the publisher.

## References

[B1] Agricola E. Bove T. Oppizzi M. Marino G. Zangrillo A. Margonato A. (2005). “Ultrasound comet-tail images”: a marker of pulmonary edema - a comparative study with wedge pressure and extravascular lung water. *Chest* 127 1690–1695. 10.1378/chest.127.5.1690 15888847

[B2] Andrikakou P. Vickraman K. Arora H. (2016). On the behaviour of lung tissue under tension and compression. *Sci. Rep.* 6 1–10. 10.1038/srep36642 27819358 PMC5098200

[B3] Assaad S. Kratzert W. B. Shelley B. Friedman M. B. Perrino A. (2018). Assessment of Pulmonary Edema: principles and Practice. *J. Cardiothorac. Vasc. Anesth.* 32 901–914. 10.1053/j.jvca.2017.08.028 29174750

[B4] Bain A. R. Ainslie P. N. Barak O. F. Hoiland R. L. Drvis I. Mijacika T. (2017). Hypercapnia is essential to reduce the cerebral oxidative metabolism during extreme apnea in humans. *J. Cereb. Blood Flow Metab.* 37 3231–3242. 10.1177/0271678X16686093 28071964 PMC5584699

[B5] Bain A. R. Drvis I. Dujic Z. MacLeod D. B. Ainslie P. N. (2018). Physiology of static breath holding in elite apneists. *Exp. Physiol.* 103 635–651. 10.1113/EP086269 29512224

[B6] Banham N. D. Lippmann J. (2019). Fatal air embolism in a breath-hold diver. *Diving Hyperb. Med.* 49 304–305. 10.28920/dhm49.4.304-305 31828750 PMC7039776

[B7] Boussuges A. Coulange M. Bessereau J. Gargne O. Ayme K. Gavarry O. (2011). Ultrasound lung comets induced by repeated breath-hold diving, a study in underwater fishermen. *Scand. J. Med. Sci. Sport.* 21 e384–92. 10.1111/j.1600-0838.2011.01319.x 21535186

[B8] Carter E. A. Mayo J. R. MacInnis M. J. McKenzie D. C. Koehle M. S. (2014). Individual susceptibility to high altitude and immersion pulmonary edema and pulmonary lymphatics. *Aviat. Sp. Environ. Med.* 85 9–14. 10.3357/ASEM.3736.2014 24479253

[B9] Chiumello D. Froio S. Colombo A. Coppola S. (2016). “Lung Ultrasound in the Critically Ill Patient” in *Topical Issues in Anesthesia and Intensive Care*, ed. Chiumello D. (Cham: Springer International Publishing), 55–67. 10.1007/978-3-319-31398-6_3

[B10] Chung S. C. S. Seccombe L. M. Jenkins C. R. Frater C. J. Ridley L. J. Peters M. J. (2010). Glossopharyngeal insufflation causes lung injury in trained breath-hold divers. *Respirology* 15 813–817. 10.1111/j.1440-1843.2010.01791.x 20546194

[B11] Cialoni D. Marabotti C. Sponsiello N. Pieri M. Balestra C. Lucchini V. (2015). Genetic predisposition to breath-hold diving-induced hemoptysis: preliminary study. *Undersea Hyperb. Med.* 42 75–83. 26094307

[B12] Cialoni D. Sponsiello N. Marabotti C. Marroni A. Pieri M. Maggiorelli F. (2012). Prevalence of acute respiratory symptoms in breath-hold divers. *Undersea Hyperb. Med.* 39 837–844. 22908840

[B13] Cortellaro F. Ceriani E. Spinelli M. Campanella C. Bossi I. Coen D. (2017). Lung ultrasound for monitoring cardiogenic pulmonary edema. *Intern. Emerg. Med.* 12 1011–1017. 10.1007/s11739-016-1510-y 27473425

[B14] Cortes-Puentes G. A. Keenan J. C. Adams A. B. Parker E. D. Dries D. J. Marini J. J. (2015). Impact of chest wall modifications and lung injury on the correspondence between airway and transpulmonary driving pressures. *Crit. Care Med.* 43 e287–95. 10.1097/CCM.0000000000001036 26186478

[B15] Dietrich C. F. Mathis G. Blaivas M. Volpicelli G. Seibel A. Wastl D. (2016). Lung B-line artefacts and their use. *J. Thorac. Dis.* 8 1356–1365. 10.21037/jtd.2016.04.55 27293860 PMC4885976

[B16] Dominelli P. B. Molgat-Seon Y. Sheel A. W. (2019). Sex Differences in the Pulmonary System Influence the Integrative Response to Exercise. *Exerc. Sport Sci. Rev.* 47 142–150. 10.1249/JES.0000000000000188 30817330

[B17] Fahlman A. Hooker S. K. Olszowka A. Bostrom B. L. Jones D. R. (2009). Estimating the effect of lung collapse and pulmonary shunt on gas exchange during breath-hold diving: the Scholander and Kooyman legacy. *Respir. Physiol. Neurobiol.* 165 28–39. 10.1016/j.resp.2008.09.013 18973832

[B18] Ferretti G. Costa M. Moroni R. Ranieri P. Butti F. Sponsiello N. (2012). Lung volumes of extreme breath-hold divers. *Sport Sci. Health* 7 55–59. 10.1007/s11332-012-0112-y

[B19] Ferrigno M. Ferretti G. Ellis A. Warkander D. Costa M. Cerretelli P. (1997). Cardiovascular changes during deep breath-hold dives in a pressure chamber. *J. Appl. Physiol.* 83 1282–1290. 10.1152/jappl.1997.83.4.1282 9338438

[B20] Fitz-Clarke J. R. (2007). Mechanics of airway and alveolar collapse in human breath-hold diving. *Respir. Physiol. Neurobiol.* 159 202–210. 10.1016/j.resp.2007.07.006 17827075

[B21] Fitz-Clarke J. R. (2018). Breath-hold diving. *Compr. Physiol.* 8 585–630. 10.1002/cphy.c160008 29687909

[B22] Frassi F. Pingitore A. Cialoni D. Picano E. (2008). Chest Sonography Detects Lung Water Accumulation in Healthy Elite Apnea Divers. *J. Am. Soc. Echocardiogr.* 21 1150–1155. 10.1016/j.echo.2008.08.001 18926391

[B23] Gouzi F. Francois G. Renard C. Jounieaux V. (2007). «Deep-purple»: un cas d’hémoptysie lors d’une plongée en apnée. *Rev. Mal. Respir.* 24 1129–1132. 10.1016/S0761-8425(07)74263-8 18176390

[B24] Heistad D. D. Abbound F. M. Eckstein J. W. (1968). Vasoconstrictor response to simulated diving in man. *J. Appl. Physiol.* 25 542–549. 10.1152/jappl.1968.25.5.542 5687360

[B25] Jambrik Z. Monti S. Coppola V. Agricola E. Mottola G. Miniati M. (2004). Usefulness of ultrasound lung comets as a nonradiologic sign of extravascular lung water. *Am. J. Cardiol.* 93 1265–1270. 10.1016/j.amjcard.2004.02.012 15135701

[B26] Kiyan E. Aktas S. Toklu A. S. (2001). Hemoptysis provoked by voluntary diaphragmatic contractions in breath-hold divers. *Chest* 120 2098–2100. 10.1378/chest.120.6.2098 11742946

[B27] Koehle M. S. Lepawsky M. McKenzie D. C. (2005). Pulmonary oedema of immersion. *Sport. Med.* 35 183–190. 10.2165/00007256-200535030-00001 15730335

[B28] Kumar M. Thompson P. D. (2019). A literature review of immersion pulmonary edema. *Phys. Sportsmed.* 47 148–151. 10.1080/00913847.2018.1546104 30403902

[B29] Lahm T. Crisostomo P. R. Markel T. A. Wang M. Weil B. R. Novotny N. M. (2008). The effects of estrogen on pulmonary artery vasoreactivity and hypoxic pulmonary vasoconstriction: potential new clinical implications for an old hormone. *Crit. Care Med.* 36 2174–2183. 10.1097/CCM.0b013e31817d1a92 18552699

[B30] Lai-Fook S. J. Rodarte J. R. (1991). Pleural pressure distribution and its relationship to lung volume and interstitial pressure. *J. Appl. Physiol.* 70 967–978. 10.1152/jappl.1991.70.3.967 2033012

[B31] Lambrechts K. Germonpré P. Charbel B. Cialoni D. Musimu P. Sponsiello N. (2011). Ultrasound lung “comets” increase after breath-hold diving. *Eur. J. Appl. Physiol.* 111 707–713. 10.1007/s00421-010-1697-y 20972574

[B32] Layoun M. E. Aboulhosn J. A. Tobis J. M. (2017). Potential role of patent foramen ovale in exacerbating hypoxemia in chronic pulmonary disease. *Texas Hear. Inst. J.* 44 189–197. 10.14503/THIJ-16-6027 28761399 PMC5505397

[B33] Lichtenstein D. (2014). Lung ultrasound in the critically ill. *Ann. Intensive Care* 4 1–12. 10.1097/MCC.0000000000000096 24401163 PMC3895677

[B34] Lindholm P. (2007). Loss of motor control and/or loss of consciousness during breath-hold competitions. *Int. J. Sports Med.* 28 295–299. 10.1055/s-2006-924361 17024640

[B35] Lindholm P. Ekborn A. Öberg D. Gennser M. (2008). Pulmonary edema and hemoptysis after breath-hold diving at residual volume. *J. Appl. Physiol.* 104 912–917. 10.1152/japplphysiol.01127.2007 18202166

[B36] Lindholm P. Lundgren C. E. (2009). The physiology and pathophysiology of human breath-hold diving. *J. Appl. Physiol.* 106 284–292. 10.1152/japplphysiol.90991.2008.-This 18974367

[B37] Lindholm P. Swenson E. R. Martínez-Jiménez S. Guo H. H. (2018). From ocean deep to mountain high: similar computed tomography findings in immersion and high-altitude pulmonary edema. *Am. J. Respir. Crit. Care Med.* 198 1088–1089. 10.1164/rccm.201803-0581IM 30044644

[B38] Linér M. H. Andersson J. P. A. (2008). Pulmonary edema after competitive breath-hold diving. *J. Appl. Physiol.* 104 986–990. 10.1152/japplphysiol.00641.2007 18218906

[B39] Lund K. L. Mahon R. T. Tanen D. A. Bakhda S. (2003). Swimming-induced pulmonary edema. *Ann. Emerg. Med.* 41 251–256. 10.1067/mem.2003.69 12548277

[B40] Magnani S. Mulliri G. Sainas G. Ghiani G. Pinna V. Sanna I. (2018). Occurrence of cardiac output decrease (via stroke volume) is more pronounced in women than in men during prolonged dry static apnea. *J. Appl. Physiol.* 124 349–355. 10.1152/japplphysiol.00991.2016 29051338

[B41] Mahon R. T. Kerr S. Amundson D. Parrish J. S. (2002). Immersion Pulmonary Edema in Special Forces Combat Swimmers. *Chest* 122 383–384. 10.1378/chest.122.1.383-a 12114391

[B42] McKnight J. C. Mulder E. Ruesch A. Kainerstorfer J. Wu J. Hakimi N. (2021). When the human brain goes diving: using NIRS to measure cerebral and systemic cardiovascular responses to deep breath-hold diving in elite freedivers. *Philos. Trans. R. Soc. B Biol. Sci.* 376. “in press.” 10.1098/rstb.2020.0349 PMC8237162 34176327

[B43] Moon R. E. Martina S. D. Peacher D. F. Potter J. F. Wester T. E. Cherry A. D. (2016). Swimming-induced pulmonary edema. *Circulation* 133 988–996. 10.1161/CIRCULATIONAHA.115.019464 26882910 PMC5127690

[B44] Mulder E. Schagatay E. (2021). Using Underwater Pulse Oximetry in Freediving to Extreme Depths to Study Risk of Hypoxic Blackout and Diving Response Phases. *Front. Physiol.* 12:651128. 10.3389/fphys.2021.651128 33868018 PMC8047056

[B45] Nitzan M. Romem A. Koppel R. (2014). Pulse oximetry: fundamentals and technology update. *Med. Devices Evid. Res.* 7 231–239. 10.2147/MDER.S47319 25031547 PMC4099100

[B46] Patrician A. Spajić B. Gasho C. Caldwell H. G. Dawkins T. Stembridge M. (2021). Temporal changes in pulmonary gas exchange efficiency when breath-hold diving below residual volume. *Exp. Physiol.* 106 1120–1133. 10.1113/EP089176 33559974

[B47] Pearn J. H. Franklin R. C. Peden A. E. (2015). Hypoxic Blackout: diagnosis. *Risks, and Prevention. Int. J. Aquat. Res. Educ.* 9 342–347. 10.25035/ijare.09.03.09

[B48] Picano E. Frassi F. Agricola E. Gligorova S. Gargani L. Mottola G. (2006). Ultrasound lung comets: a clinically useful sign of extravascular lung water. *J. Am. Soc. Echocardiogr.* 19 356–363. 10.1016/j.echo.2005.05.019 16500505

[B49] Picano E. Pellikka P. A. (2016). Ultrasound of extravascular lung water: a new standard for pulmonary congestion. *Eur. Heart J.* 37 2097–2104. 10.1093/eurheartj/ehw164 27174289 PMC4946750

[B50] Plataki M. Hubmayr R. D. (2010). The physical basis of ventilator-induced lung injury. *Expert. Rev. Respir. Med.* 4 373–385. 10.1586/ers.10.28 20524920 PMC2904955

[B51] Pratali L. Rimoldi S. F. Rexhaj E. Hutter D. Faita F. Salmoń C. S. (2012). Exercise induces rapid interstitial lung water accumulation in patients with chronic mountain sickness. *Chest* 141 953–958. 10.1378/chest.11-0084 21885723

[B52] Ross E. M. Matteucci M. J. Shepherd M. Barker M. Orr L. (2013). Measuring arterial oxygenation in a high altitude field environment: comparing portable pulse oximetry with blood gas analysis. *Wilderness Environ. Med.* 24 112–117. 10.1016/j.wem.2012.11.009 23434169

[B53] Schagatay E. (2009). Predicting performance in competitive apnoea diving. Part I: static apnoea. *Diving Hyperb. Med.* 39 88–99. 22753202

[B54] Schagatay E. (2010). Predicting performance in competitive apnea diving. Part II: dynamic apnea. *Diving Hyperb. Med.* 40 11–22. 23111834

[B55] Schagatay E. (2011). Predicting performance in competitive apnea diving. Part III: depth. *Diving Hyperb. Med.* 41 216–228. 22183699

[B56] Schagatay E. Lodin-Sundström A. Schagatay F. Engan H. (2015). “Can SaO2 measurements during recovery be used to detect lung barotrauma in freedivers?” in *41st Congress of the European Underwater & Baromedical Society (EUBS)*, eds Balestra C. van den Brink A. van Hulst R. (Amsterdam: European Underwater and Baromedical Society).

[B57] Scherhag A. Pfleger S. Grosselfinger R. Borggrefe M. (2005). Does competitive apnea diving have a long-term risk? Cardiopulmonary findings in breath-hold divers. *Clin. J. Sport Med.* 15 95–97. 10.1097/01.jsm.0000157650.07492.c5 15782054

[B58] Schipke J. D. Lemaitre F. Cleveland S. Tetzlaff K. (2019). Effects of Breath-Hold Deep Diving on the Pulmonary System. *Respiration* 97 476–483. 10.1159/000495757 30783070

[B59] Soldati G. Demi M. Demi L. (2019). Ultrasound patterns of pulmonary edema. *J. Threat. Taxa* 7:16. 10.21037/atm.2019.01.49 31032297 PMC6462619

[B60] Soldati G. Demi M. Inchingolo R. Smargiassi A. Demi L. (2016). On the physical basis of pulmonary sonographic interstitial syndrome. *J. Ultrasound Med.* 35 2075–2086. 10.7863/ultra.15.08023 27503755

[B61] Sylvester J. T. Shimoda L. A. Aaronson P. I. Ward J. P. T. (2012). Hypoxic Pulmonary Vasoconstriction. *Physiol. Rev.* 92 367–520. 10.1152/physrev.00041.2010 22298659 PMC9469196

[B62] Vestin P. (2015). *Official Summary of the Autopsy Reports Following the Death of Nicholas Mevoli.* Switzerland: AIDA International.

[B63] Volpicelli G. Elbarbary M. Blaivas M. Lichtenstein D. A. Mathis G. Kirkpatrick A. W. (2012). International evidence-based recommendations for point-of-care lung ultrasound. *Intensive Care Med.* 38 577–91. 10.1007/s00134-012-2513-4 22392031

[B64] Wang Y. Shen Z. Lu X. Zhen Y. Li H. (2018). Sensitivity and specificity of ultrasound for the diagnosis of acute pulmonary edema: a systematic review and meta-analysis. *Med. Ultrason.* 20 32–36. 10.11152/mu-1223 29400365

[B65] West J. B. (2004). Vulnerability of Pulmonary Capillaries during Exercise. *Exerc. Sport Sci. Rev.* 32 24–30. 10.1097/00003677-200401000-00006 14748546

[B66] West J. B. Tsukimoto K. Mathieu-Costello O. Prediletto R. (1991). Stress failure in pulmonary capillaries. *J. Appl. Physiol.* 70 1731–1742. 10.1152/jappl.1991.70.4.1731 2055852

[B67] Willie C. K. Ainslie P. N. Drvis I. MacLeod D. B. Bain A. R. Madden D. (2015). Regulation of brain blood flow and oxygen delivery in elite breath-hold divers. *J. Cereb. Blood Flow Metab.* 35 66–73. 10.1038/jcbfm.2014.170 25370857 PMC4294396

[B68] Wilson B. J. Cowan H. J. Lord J. A. Zuege D. J. Zygun D. A. (2010). The accuracy of pulse oximetry in emergency department patients with severe sepsis and septic shock: a retrospective cohort study. *BMC Emerg. Med.* 10:9. 10.1186/1471-227X-10-9 20444248 PMC2876142

